# Supplementary material for: An RNA excited conformational state at atomic resolution
Source: Nat Commun. 2023 Dec 19;14:8432. doi: 10.1038/s41467-023-43673-6 (PMC10730710; doi:10.1038/s41467-023-43673-6)
Supplement: Supplementary file 1 — Supplementary Information [file 41467_2023_43673_MOESM1_ESM.pdf]

Supplementary Information

An RNA excited conformational state at atomic resolution

Ainan Geng[1], Laura Ganser[1 ¥], Rohit Roy[2], Honglue Shi[3†], Supriya Pratihar[4], David A. Case[5], and Hashim M. Al-Hashimi[4*]

[1]Department of Biochemistry, Duke University School of Medicine, Durham, NC 27710, USA

[2]Center for Genomic and Computational Biology, Duke University School of Medicine, Durham, NC 27710, USA

[3]Department of Chemistry, Duke University, Durham, NC 27708, USA

[4]Department of Biochemistry and Molecular Biophysics, Columbia University, New York, New York 10032, United States.

[5]Department of Chemistry and Chemical Biology, Rutgers University, Piscataway, NJ 08854, USA

¥ Current Addresses: Department of Biophysics, Johns Hopkins University, Baltimore, MD 21218, USA

† Current Addresses: Innovative Genomics Institute, University of California, Berkeley, CA 94720, USA

* To whom correspondence should be addressed.

Email: ha2639@cumc.columbia.edu

Supplementary Figures

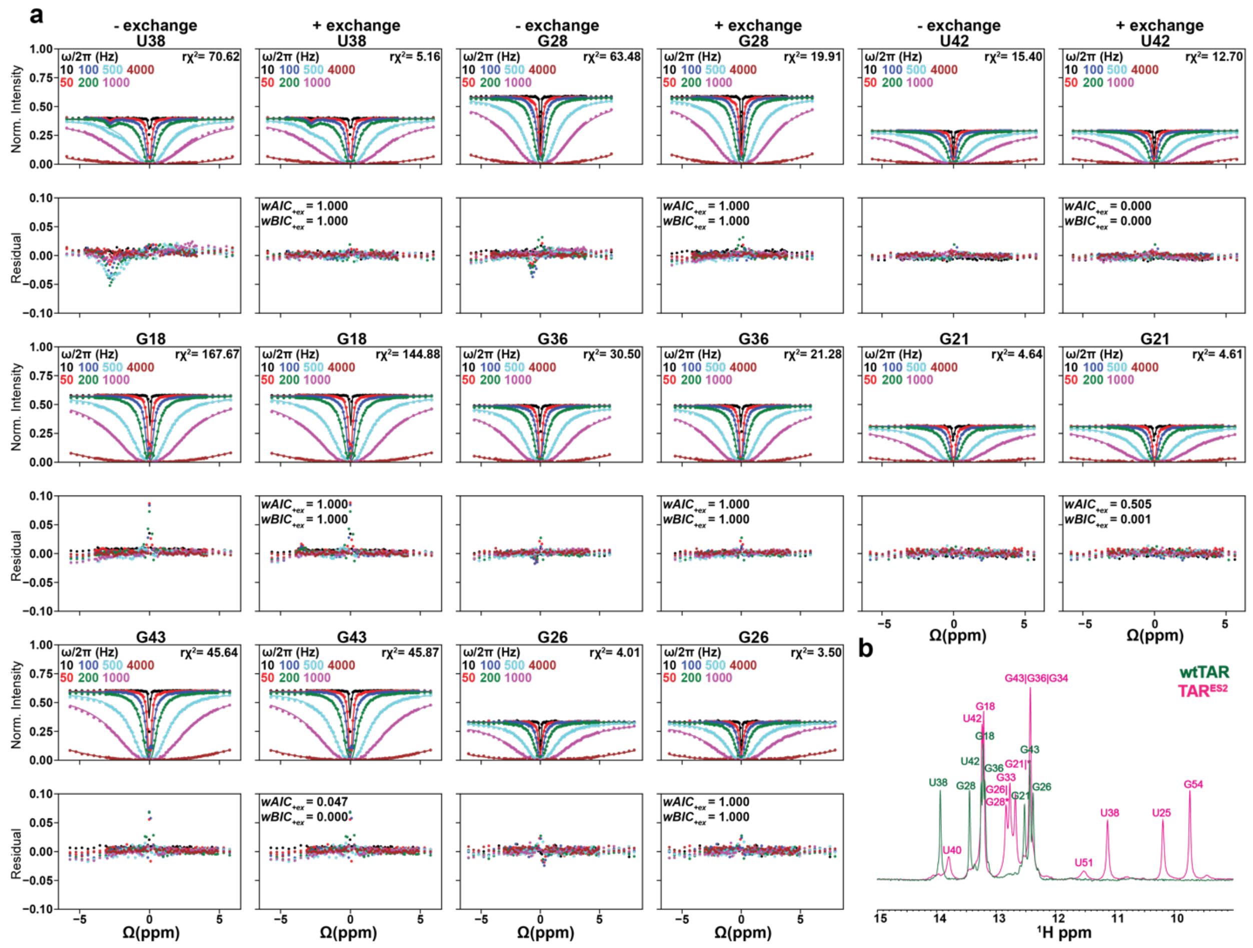

Supplementary Figure 1. ^{1}H CEST profiles measured on wtTAR at 25°C. (**a**) ^{1}H CEST profiles measured in wtTAR at 25°C. ^{1}H CEST data were fit to a two-state model with (+ex) or without (-ex, $k_{ex} = \Delta\omega = p_{ES} = 0$) exchange using Bloch–McConnell equations. Model selection (+ex or -ex) was determined based on the reduced chi-square ($r\chi^2$), Akaike's (wAIC), and Bayesian (wBIC) information criterion weights (Methods). Also shown are corresponding residual plots (normalized experimental intensity - fitted normalized intensity). The error bars for the ^{1}H CEST profile are smaller than the data point and were derived from the standard deviation of three measurements of peak intensity with zero relaxation delay. RF field powers used are color-coded. (**b**) Overlay of 1D ^{1}H imino spectra measured for wtTAR and TAR^{ES2}, showing differences in the ^{1}H chemical shifts.

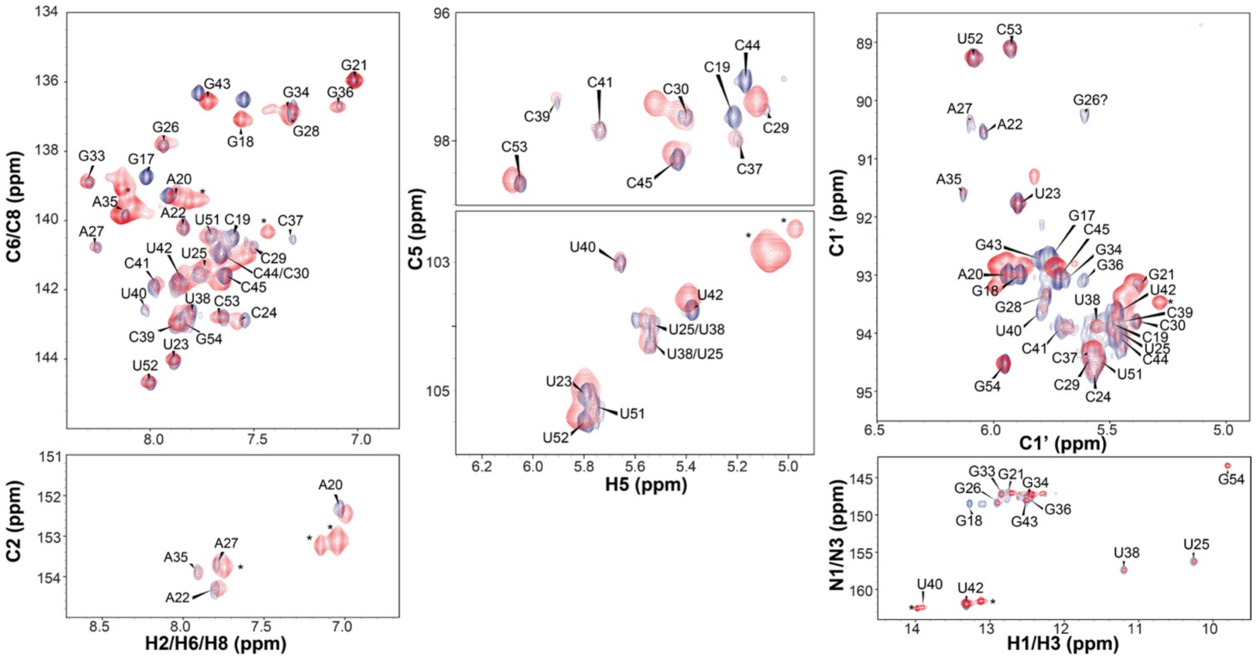

Supplementary Figure 2. The assignment of TAR^{ES2} and E-TAR^{ES2}. Overlay of 2D ^1H–^{15}N and ^1H–^{13}C HSQC spectra of E-TAR^{ES2} (in red) on corresponding spectra of TAR^{ES2} (in blue). Resonances belonging to the elongated A-U helix are indicated using an asterisk. Ambiguous assignments have more than one label.

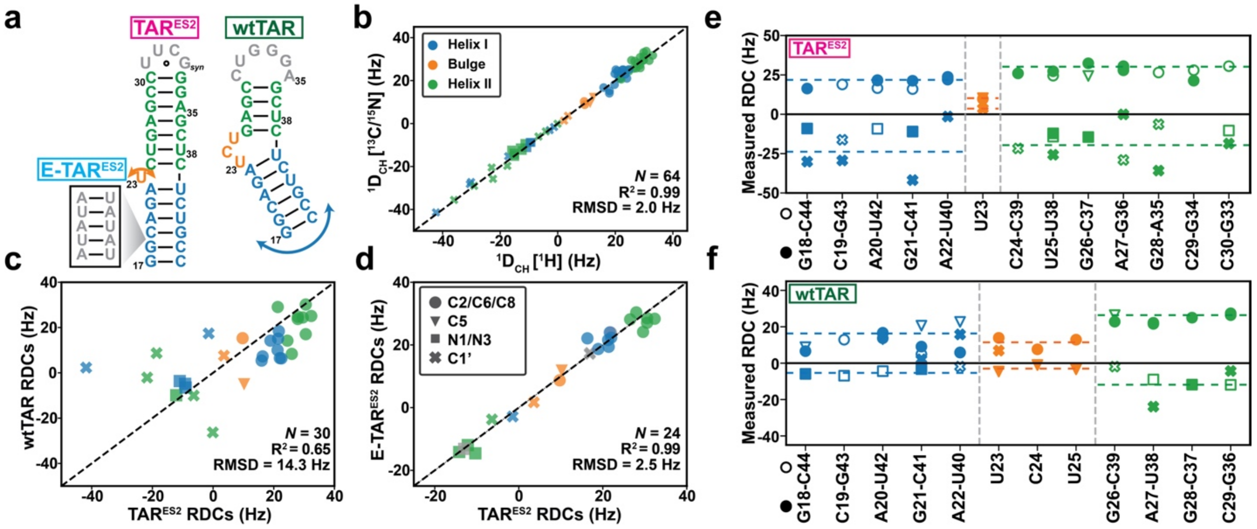

Supplementary Figure 3. Measurement of RDCs. (**a**) Secondary structure of TAR^{ES2} and its elongated counterpart, E-TAR^{ES2}. For comparison the secondary structure of the wtTAR is also shown. (**b**) Comparison of C–H and N–H splittings (C8/6/2H8/6/2 (circle), C5H5 (triangle), C1′H1′ (cross), and N1/3H1/3 (square)) measured using two frequency-based experiments (Methods). The two experiments yield splittings along either the direct (^{1}H) or indirect (^{13}C/^{15}N) dimensions. The agreement between the two sets of measurements was used to estimate the RDC uncertainty of ~ 2.0 Hz. (**c-d**) Correlation plot comparing RDCs measured in TAR^{ES2} with values measured in (**c**) wtTAR[1] and (**d**) E-TAR^{ES2}. Also shown are the number of data points (*N*), R^2 and the RMSD between measured and predicted RDCs. (**e-f**) Plot showing individual site- and residue-specific RDCs measured in (**e**) TAR^{ES2} and (**f**) wtTAR. All RDCs are color-coded according to different motifs, as shown in (**a**).

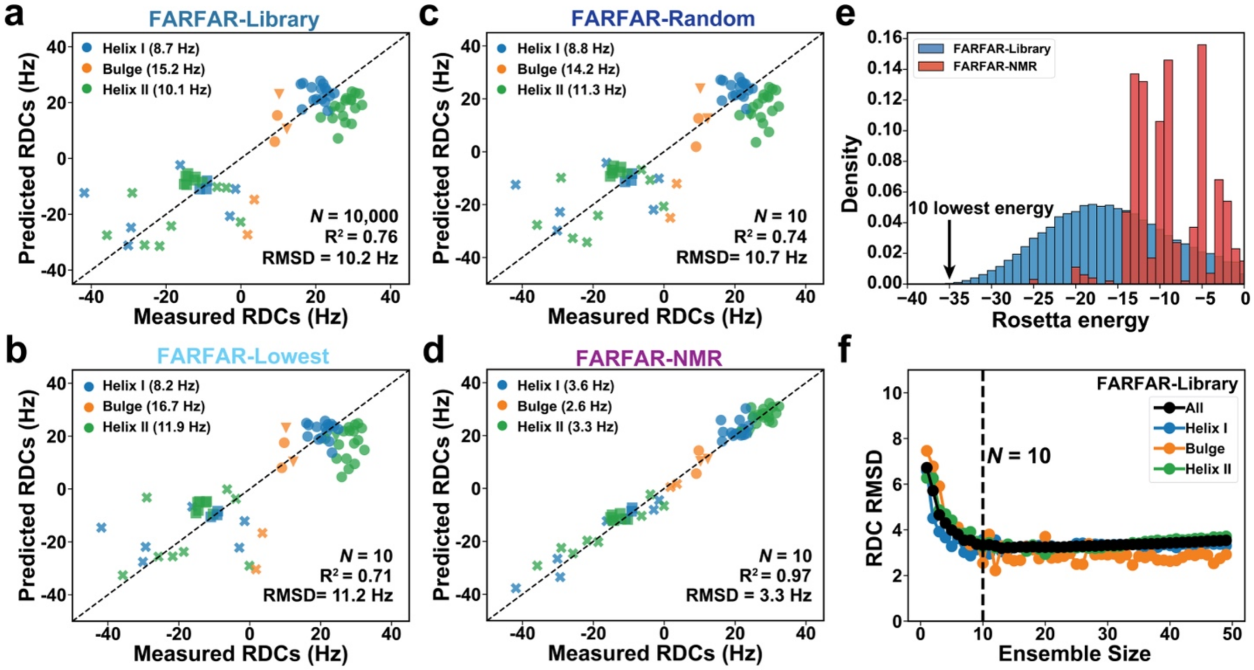

Supplementary Figure 4. Determining the FARFAR TAR^{ES2} conformational ensemble. (**a-d**) Comparison of the agreement between the measured and predicted RDCs using (**a**) FARFAR-library (*N* = 10,000) (**b**), FARFAR-Lowest (*N* = 10), (**c**) FARFAR-Random (*N* = 10) and (**d**) FARFAR-NMR (*N* = 10). (**e**) The Rosetta energy distribution of the FARFAR-library (blue, *N* = 10,000) and FARFAR-NMR ensemble (red, *N* = 1,000). The Rosetta energy for the 10 lowest energy scores is highlighted using an arrow. *N* represents the number of conformers in the library or the ensemble. (**f**) RMSD between measured and predicted RDCs (TAR^{ES2} + E-TAR^{ES2}) as a function of ensemble size (*N*) during SAS for the FARFAR-library (Methods). The selected ensemble size *N* = 10 is indicated using a vertical dashed line. All RDCs are color-coded according to different motifs, as shown in Fig. 3a.

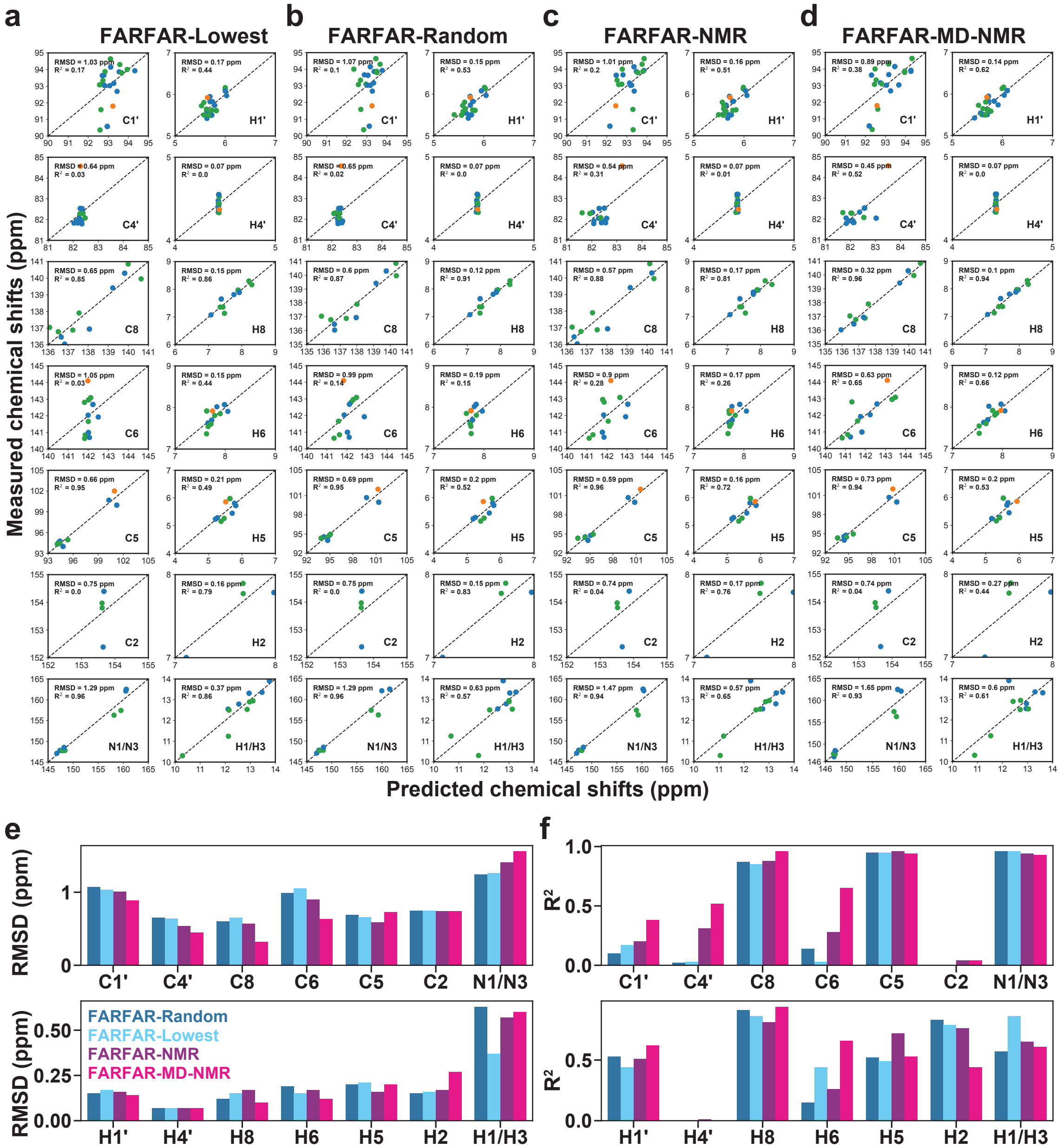

Supplementary Figure 5. Cross-validation of the TAR^{ES2} conformational ensembles using ^{13}C, ^{15}N, and ^{1}H chemical shifts measured in TAR^{ES2}. (**a-d**) Comparison of measured and predicted ^{13}C, ^{15}N, and ^{1}H chemical shifts for (**a**) FARFAR-Lowest (*N* = 10), (**b**) FARFAR-Random (*N* = 10), (**c**) FARFAR-NMR (*N* = 10), and (**d**) FARFAR-MD-NMR (*N* = 10) with color coded motifs based on Fig. 3a. *N* represents the number of conformers in the ensemble. (**e-f**) The RMSD (**e**) and R^2 (**f**) between measured and predicted ^{13}C/^{15}N (top) and ^{1}H (below) chemical shifts for the FARFAR-Random (deep blue), FARFAR-Lowest (light blue), FARFAR-NMR (purple), and FARFAR-MD-NMR (magenta) ensembles.

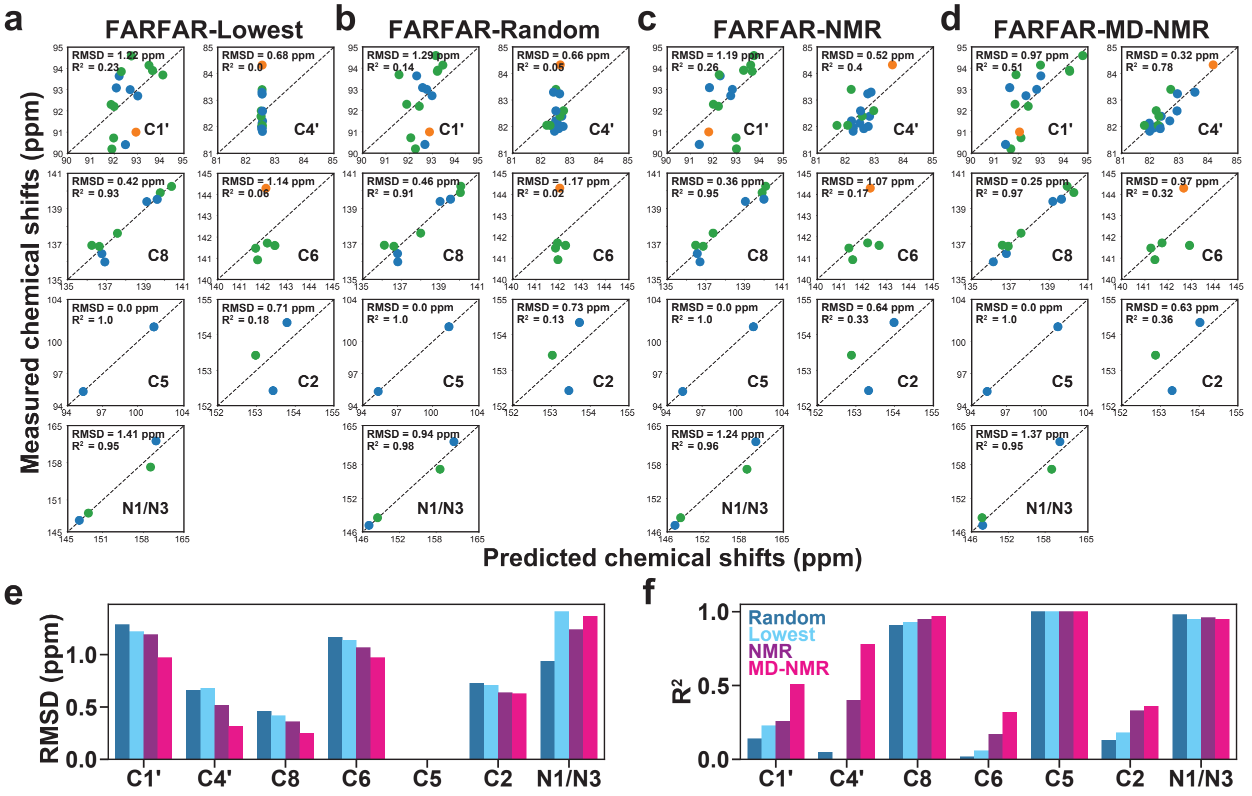

Supplementary Figure 6. Cross-validation of the TAR^{ES2} conformational ensembles using ^{13}C and ^{15}N chemical shifts measured in wtTAR ES2. (**a-d**) Comparison of measured and predicted ^{13}C, ^{15}N chemical shifts for (**a**) FARFAR-Lowest (*N* = 10), (**b**) FARFAR-Random (*N* = 10), (**c**) FARFAR-NMR (*N* = 10), and (**d**) FARFAR-MD-NMR (*N* = 10) with color coded motifs based on Fig. 3a. *N* represents the number of conformers in the ensemble. (**e-f**) RMSD (**e**) and R^2 (**f**) between measured and predicted ^{13}C/^{15}N (top) chemical shifts for the FARFAR-Random (deep blue), FARFAR-Lowest (light blue), FARFAR-NMR (purple), and FARFAR-MD-NMR (magenta) ensembles.

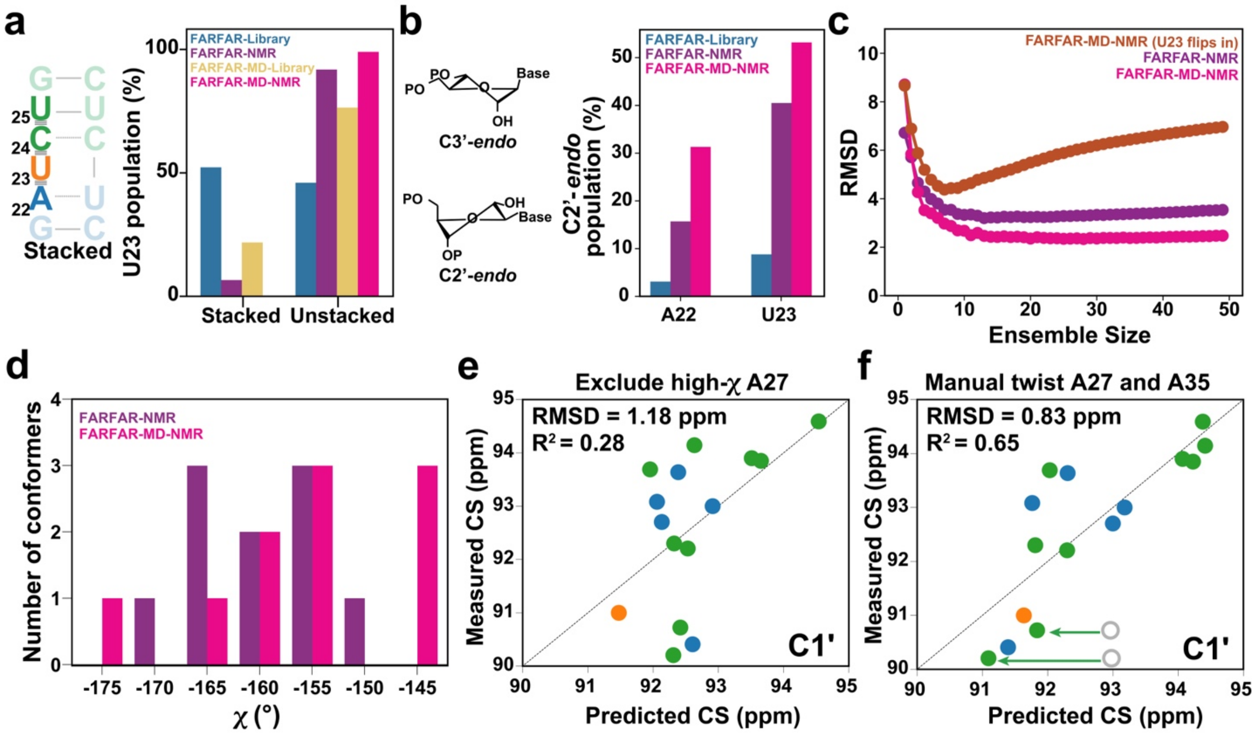

Supplementary Figure 7. Testing features of the TAR^{ES2} ensemble. (**a**) Population of U23 stacked intra-helically in the FARFAR-Library (deep blue), FARFAR-NMR (N = 10 × 100 = 1000, purple), FARFAR-MD-Library (yellow), and FARFAR-MD-NMR (N = 10 × 100 = 1000, magenta). (**b**) Population of C2'-*endo* pucker for A22 and U23 in the FARFAR-Library (deep blue), FARFAR-NMR (N = 10 × 100 = 1000, purple), and FARFAR-MD-NMR (N = 10 × 100 = 1000, magenta). Structural features in the FARFAR-Library and FARFAR-MD-Library libraries were assessed by randomly selecting N = 1000 conformers from the library. (**c**) RDC RMSD as a function of ensemble size (N) during SAS when using the FARFAR (purple) and FARFAR-MD (magenta) libraries and for the FARFAR-MD-Library (brown) after removing conformations in which U23 is flipped out. (**d**) The χ-angles distribution of the FARFAR-NMR (N = 10, purple) and FARFAR-MD-NMR ensemble (N = 10, magenta). N in (a-d) represents the number of conformers in the library or the ensemble. (**e**) Comparison of the measured versus predicted C1' chemical shifts for FARFAR-MD-NMR ensemble after removing conformers with high A27 χ-angles (−142° to −146°). (**f**) Comparison of the measured versus predicted C1' chemical shifts for FARFAR-NMR ensemble before (hollow symbols) and after (solid symbols) adjusting (Methods) the χ-angles for A27 and A35. The ES2 experimental chemical shifts were obtained from RD measurements on wtTAR[2,3]. The chemical shifts are color-coded according to different motifs, as shown in Fig. 3a.

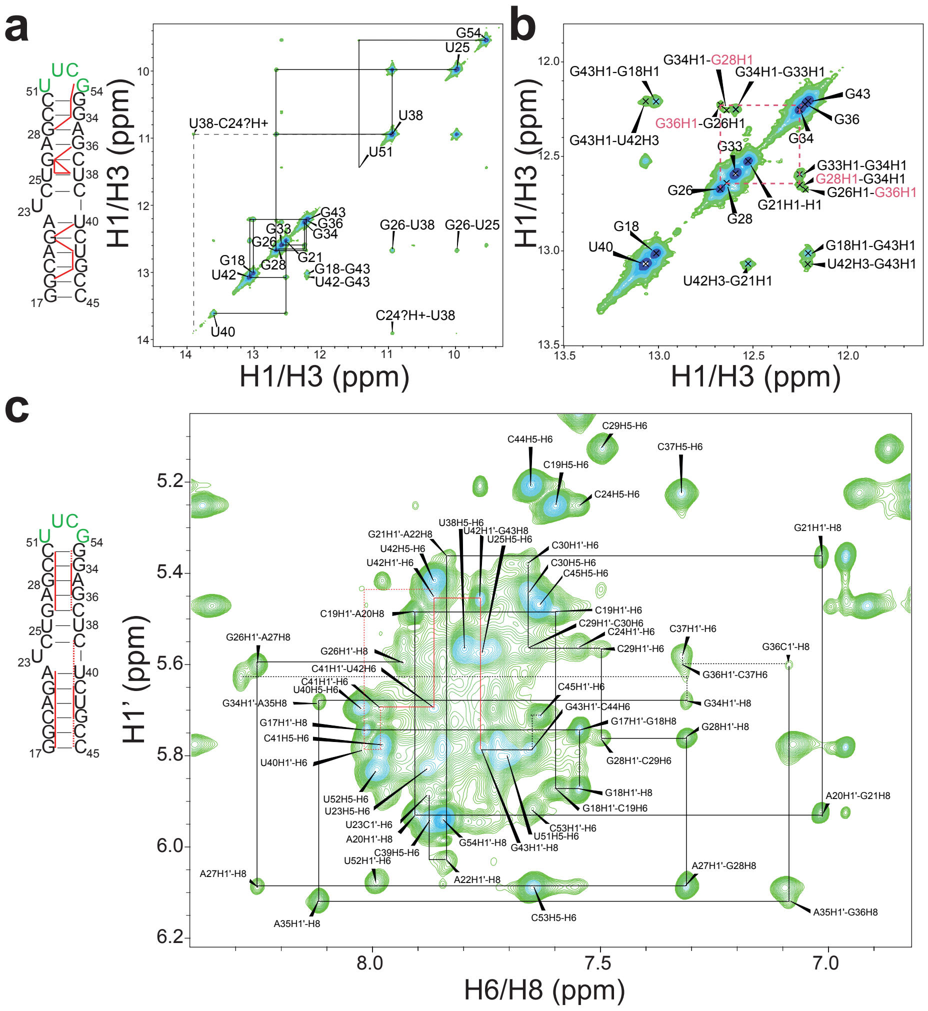

Supplementary Figure 8. 2D ^{1}H-^{1}H NOESY spectra of TAR^{ES2}. (**a**) the exchangeable NOE walk measured on a 900 MHz spectrometer at 5 ºC in 10% D_2O with mixing time 200 ms. The imino resonances and NOE-based distance connectivity, highlighted in red on the secondary structure, show formation of U25-U38 wobble, G26-C37 Watson-Crick bp, and two Watson-Crick G_{anti}-A_{anti} mismatches. (**b**) A zoomed-in view of the exchangeable 2D NOESY spectrum showing connectivity belonging to the A-G mismatches. The G28-H1 and G36-H1 (highlighted in red) imino resonances fall in the canonical Watson-Crick G-H1 region. The red dash line highlights the G26H1-G36H1 and G28H1-G34H1 cross-peaks. (**c**) the non-exchangeable NOE walk measured on a 600 MHz spectrometer at 25 ºC in 100% D_2O with mixing time 200 ms[4]. Also shown is the corresponding distance-based connectivity highlighted in red on the secondary structure. The intra- and inter-nucleotide H8/6-H1' NOEs were interrupted at U23 supporting that it partially adopts a flipped out conformation. The strong H1'-H8 cross for G54 indicates a *syn* conformation. We did not observe evidence for a sheared conformation for the A-G mismatches including upfield shifted G-H1 (9.5-10.5 ppm) within the mismatch (G28 and G36) and upfield shifted H1' (~4.3 ppm) for the neighboring residue (G34) on the 3'-end of the tandem GA mismatches[5-7].

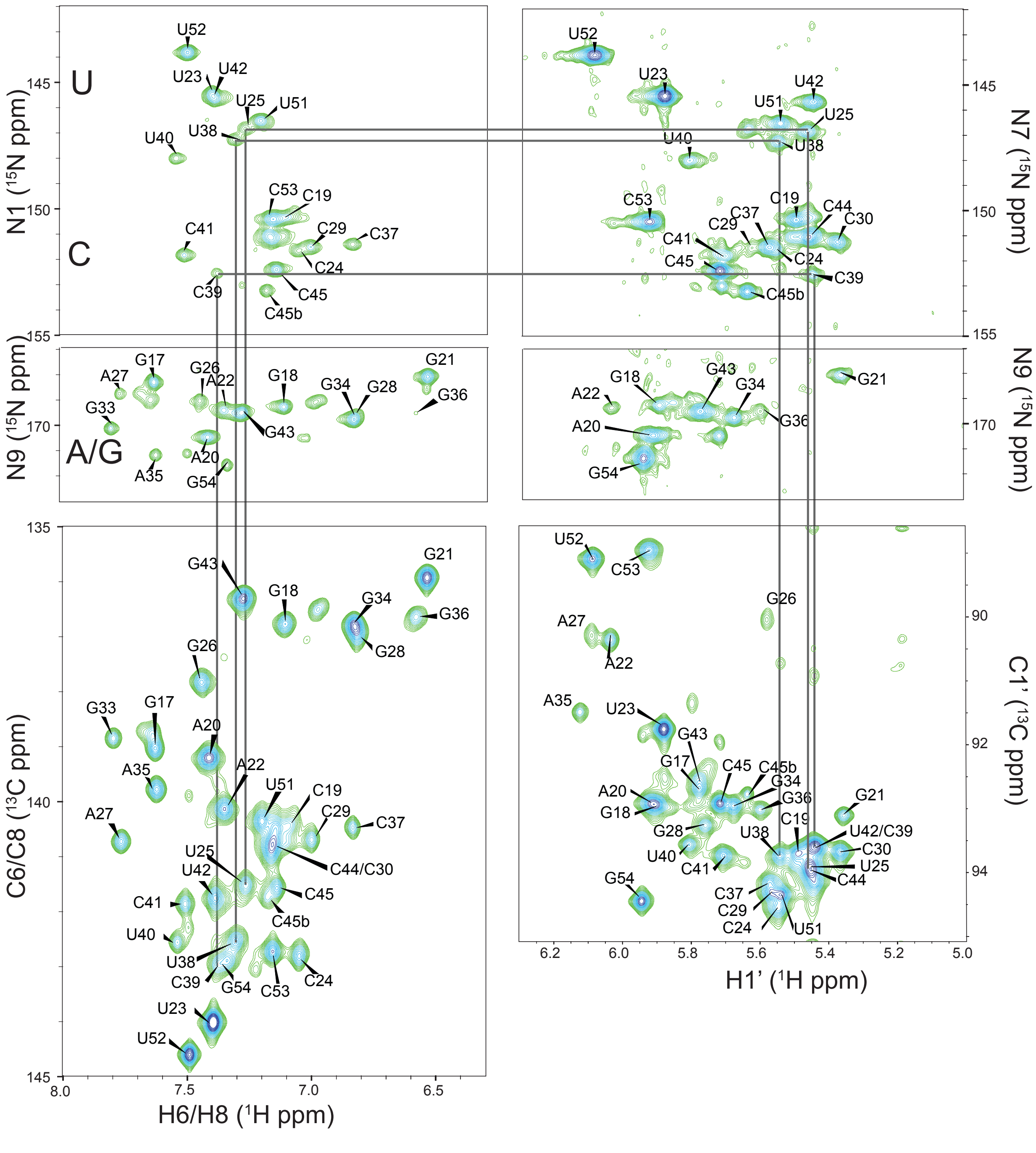

Supplementary Figure. 9 2D HCN spectra of uniformly labeled TAR^{ES2} at 15°C recorded at 600 MHz. The connectivity associated with the updated assignments are highlighted in grey.

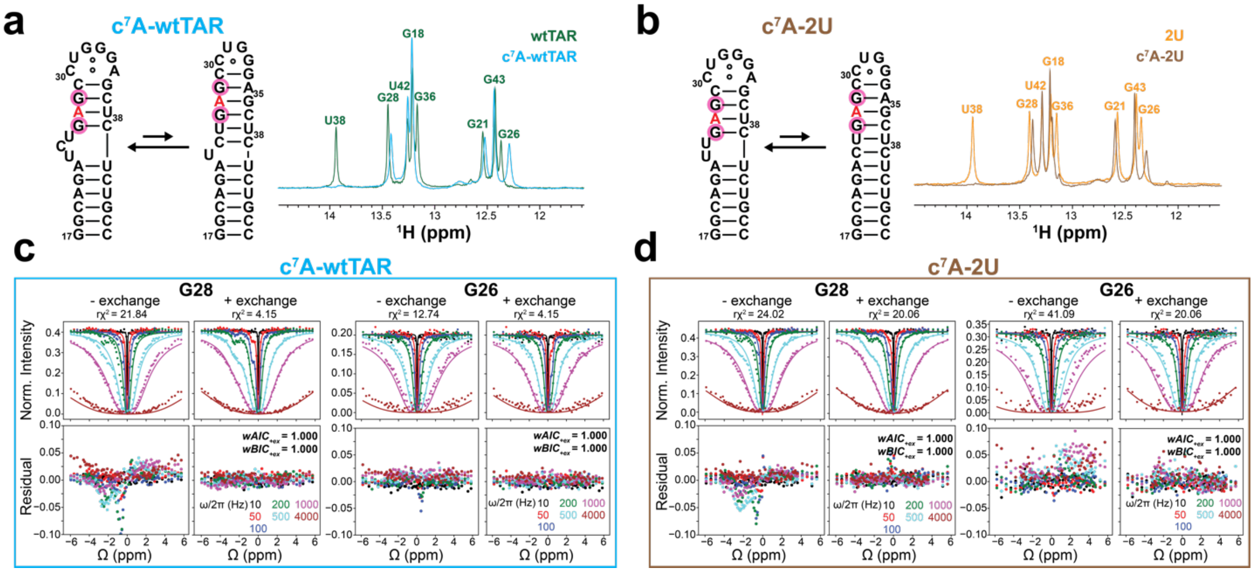

Supplementary Figure 10. c^7A preserves the GS to ES2 exchange dynamic in different sequence contexts. (**a-b**) Comparison of ^{1}H 1D spectra of the imino region of (**a**) wtTAR (green) and c^7A wtTAR (blue), and (**b**) 2U (orange) and c^7A 2U (brown); the modified residues are highlighted in red, and residues showing exchange contributions to ES2 in the ^{1}H CEST experiment are circled in pink. The absence of U38-H3 peaks is in line with prior studies in DNA in which significant line broadening was observed at c^7A modified and neighboring base pairs[8]. (**c-d**) ^{1}H CEST profiles measured in (**c**) c^7A wtTAR and (**d**) c^7A 2U at 25°C. A new excited state with Δω of ~ -2.6 ppm was observed at G28 in the tandem AG mismatch, which could be attributed to a protonated A_{syn}^{+}-G_{anti} conformation. ^{1}H CEST data measured on G28 were fit to a three-state model with (+ex) or without (-ex, $k_{ex} = \Delta\omega = p_{ES} = 0$) exchange using Bloch–McConnell equations. The G26 ^{1}H CEST profile was fitted to a two-state model in c^7A wtTAR and a three-state model in c^7A 2U. Model selection (+ex or -ex) was determined based on the reduced chi-square ($r\chi^2$), Akaike's (wAIC), and Bayesian (wBIC) information criterion weights (Methods). Also shown are corresponding residual plots (normalized experimental intensity - fitted normalized intensity). The error bars for the ^{1}H CEST profile are smaller than the data point and were derived from the standard deviation of three measurements of peak intensity with zero relaxation delay. RF field powers used are color-coded.

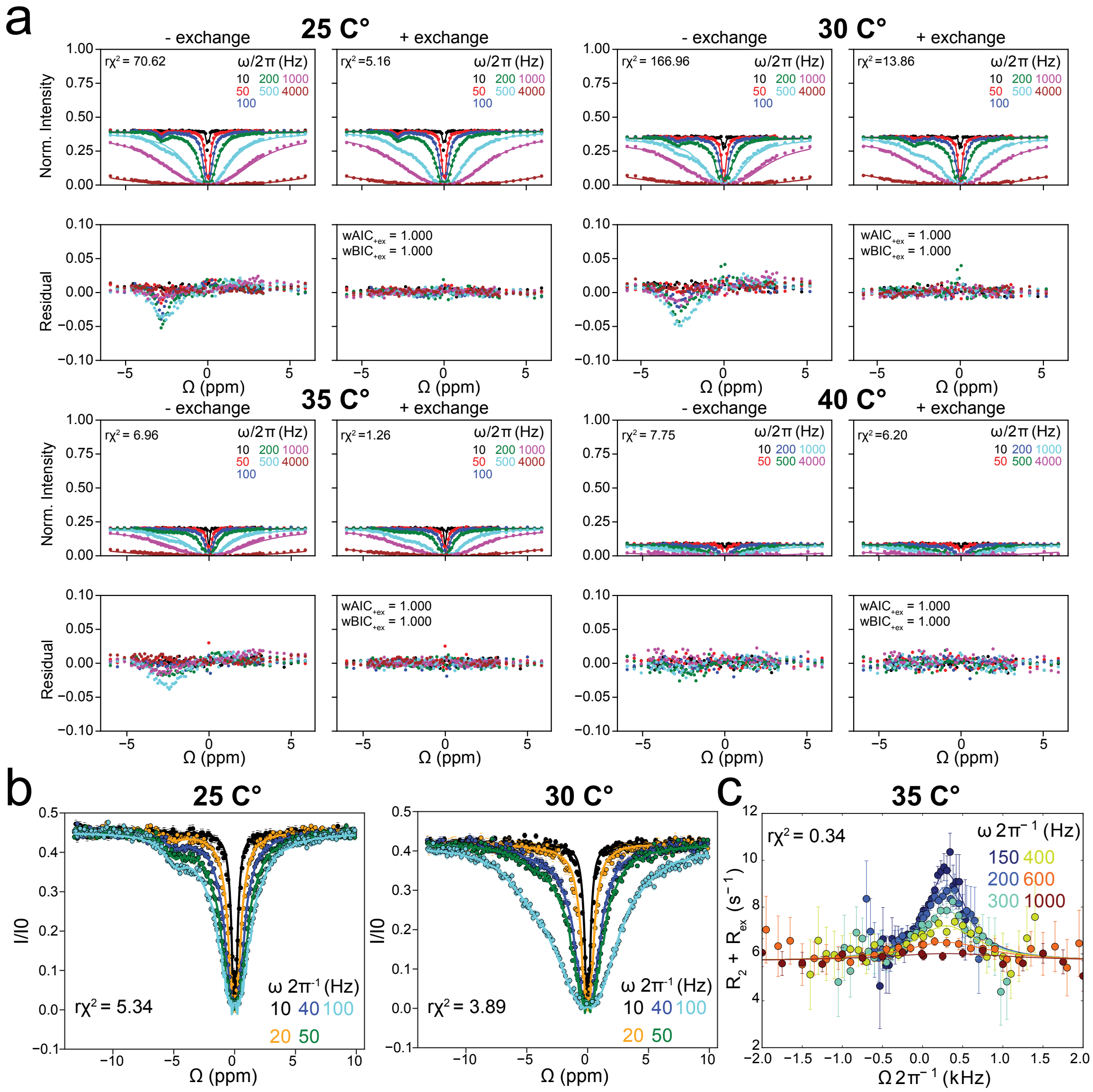

Supplementary Figure 11. Temperature-dependent wtTAR exchange profiles. (**a**) ^{1}H CEST profiles measured on U38-H3 as a function of temperature. (25°C, 30°C, 35°C, and 40°C). ^{1}H CEST data were fit to a two-state model with (+ex) or without (-ex) exchange using Bloch–McConnell equations. Model selection (+ex or -ex) was determined based on rχ^2, Akaike's (wAIC), and Bayesian (wBIC) information criterion weights (Methods). Also shown are corresponding residual plots (normalized experimental intensity - fitted normalized intensity). The error bars for the ^{1}H CEST profile are smaller than the data point and were derived from the standard deviation of three measurements of peak intensity with zero relaxation delay. (**b**) ^{15}N CEST profiles measured on U38-N3 at 25°C and 30°C. Data were fit with a two-state model using Bloch–McConnell equations and denoted as solid lines. The error bars for ^{15}N CEST were derived from the standard deviation of three measurements of peak intensity with zero relaxation delay. (**c**) Off-resonance $R_{1\rho}$ RD profiles measured on U38-N3 at 35°C. Data were fit with a two-state model using Bloch–McConnell equations and denoted as solid lines. Errors in R_2 + R_{ex} were determined by propagating the error in fitted monoexponential decay curves and experimental signal to noise through a Monte Carlo procedure, as previously described[9]. RF powers used for ^{1}H and ^{15}N CEST and spin-lock powers used for $R_{1\rho}$ are color coded.

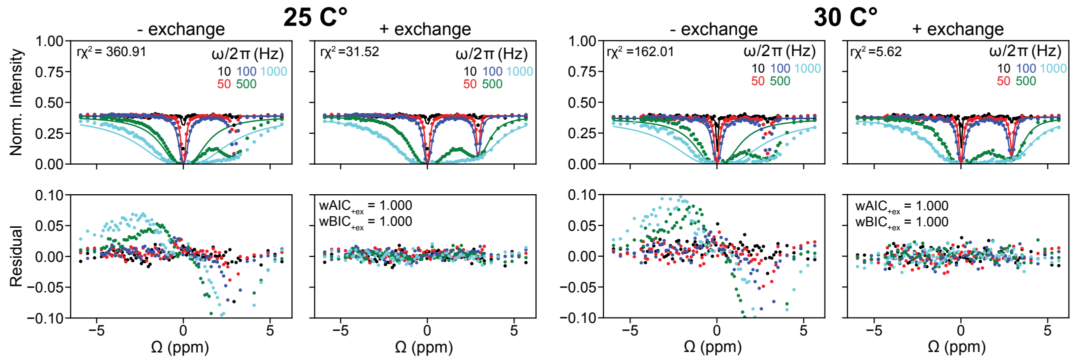

Supplementary Figure. 12 Temperature-dependent TAR^{ES2} ^{1}H CEST profiles. ^{1}H CEST profiles measured for TAR^{ES2} U38-H3 as a function of temperature. ^{1}H CEST data were fit to a two-state model with (+ex) or without (-ex) exchange using Bloch–McConnell equations. Also shown are corresponding residual plots (normalized experimental intensity - fitted normalized intensity). Model selection (+ex or -ex) was determined based on the reduced chi-square ($r\chi^2$), Akaike's (wAIC), and Bayesian (wBIC) information criterion weights (Methods). The error was derived from the standard deviation of three measurements of peak intensity with zero relaxation delay. RF powers used for ^{1}H CEST are color coded.

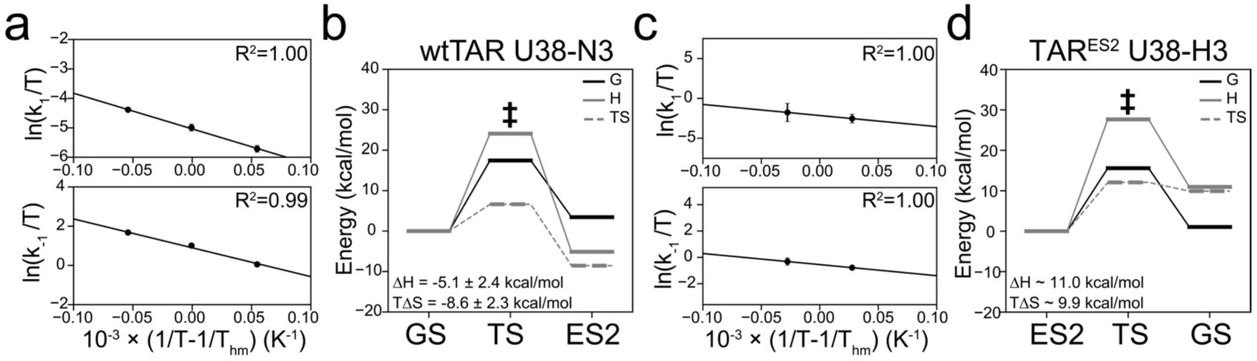

Supplementary Figure 13. Kinetic-thermodynamic analysis of ground-to-excited and excited-to-ground state transitions. (**a**) Temperature-dependent forward (k_1) and reverse (k_{-1}) rate constants for the GS to ES2 exchange obtained using ^{15}N CEST at 25°C and 30°C and ^{15}N $R_{1\rho}$ at 35°C for U38-N3 in wtTAR. (**b**) The kinetic-thermodynamic profile for exchange between the GS and ES2 in wtTAR via a transition state (‡), showing activation and net free energy (G), enthalpy (H), and entropy (TS) changes (with GS referenced to 0). (**c**) Temperature-dependent forward (k_1) and reverse (k_{-1}) rate constants for the ES2 to GS exchange obtained using ^{1}H CEST for U38-H3 in the TAR^{ES2} mutant. (**d**) The kinetic-thermodynamic profile for exchange between the ES2 and GS in the TAR^{ES2} mutant via a transition state (‡), showing activation and net free energy (G), enthalpy (H), and entropy (TS) changes (with ES2 referenced to 0). Error bars in (b) and (d) for k_1 and k_{-1} were determined by propagating the errors in exchange parameters obtained from 2-state fits of the ^{1}H CEST, ^{15}N CEST or $R_{1\rho}$ profile for U38 to the Bloch-McConnell equations (Methods).

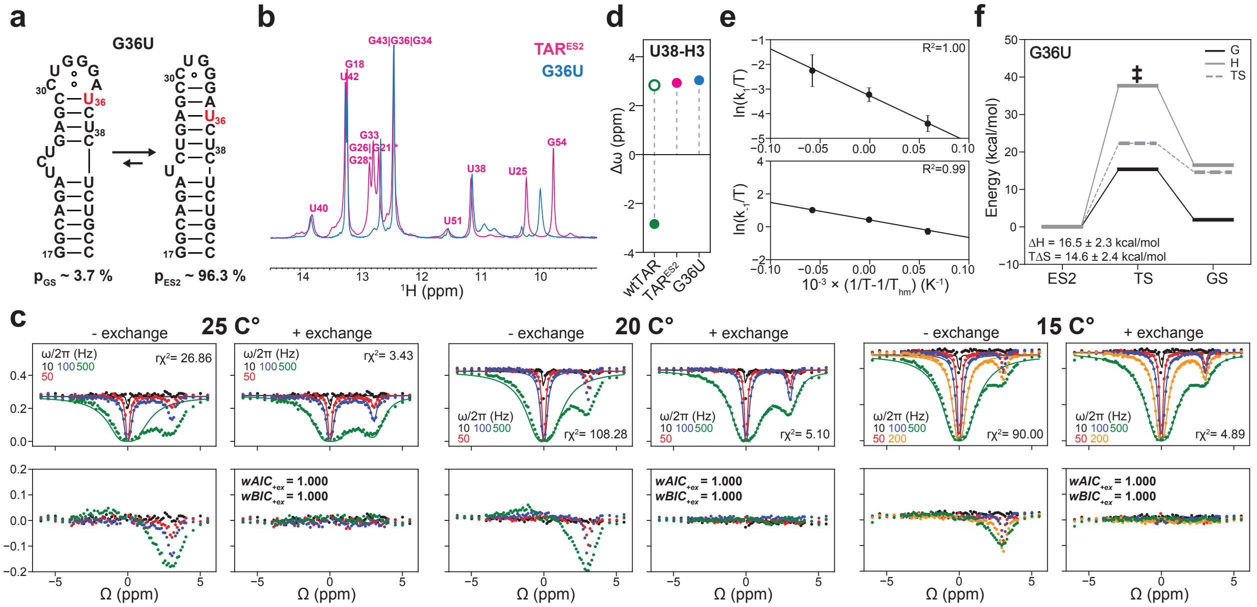

Supplementary Figure 14. Kinetic-thermodynamic analysis of the excited-to-ground state transition. (**a**) Chemical exchange between the GS and ES in the G36U mutant. Also shown are the populations (p_{GS} and p_{ES2}) and exchange rate constant deduced using ^{1}H CEST experiment at 25°C. The mutation site is colored in red. (**b**) Overlay of 1D ^{1}H imino spectra measured for G36U and TAR^{ES2}, showing similar ^{1}H chemical shifts. (**c**) Temperature-dependent G36U exchange profiles. ^{1}H CEST profiles measured on U38-H3 as a function of temperature (15°C, 20°C, and 25°C). ^{1}H CEST data were fit to a two-state model with (+ex) or without (-ex) exchange using Bloch–McConnell equations. Model selection (+ex or -ex) was determined based on $r\chi^2$, Akaike's (wAIC), and Bayesian (wBIC) information criterion weights (Methods). Also shown are corresponding residual plots (normalized experimental intensity - fitted normalized intensity). The error bars for the ^{1}H CEST profile are smaller than the data point and were derived from the standard deviation of three measurements of peak intensity with zero relaxation delay. (**d**) Comparison of the ^{1}H chemical shifts difference ($\Delta\omega = \omega_{ES} - \omega_{GS}$) measured on wtTAR (green), TAR^{ES2} (pink), and G36U (blue). The open circle denotes the absolute value of wtTAR Δω. In all cases, the uncertainty in $\Delta\omega < 0.01$ ppm. (**e**)Temperature-dependent forward (k_1) and reverse (k_{-1}) rate constants for the ES2 to GS exchange obtained using ^{1}H CEST in G36U. Error bars in k_1 and k_{-1} were determined by propagating the errors of exchange parameters obtained from 2-state fits of the ^{1}H CEST profile for U38-H3 to the Bloch-McConnell equations. (**f**) The kinetic-thermodynamic profile for exchange between the ES2 and GS in G36U via a transition state (‡), showing activation and net free energy (G), enthalpy (H), and entropy (TS) changes with ES2 referenced to 0.

Supplementary Tables

Supplementary Table 1. RDCs measured in TAR^{ES2} and E-TAR^{ES2} at 25˚C in NMR.

Residue	Bond Vector	Measured RDC (Hz)	
		TAR^{ES2}	E-TAR^{ES2}
G18	C1'-H1'	-30.1	NA
G18	C8-H8	16.3	23
G18	N1-H1	-9	NA
C19	C1'-H1'	-29.4	NA
A20	C2-H2	21.7	25
A20	C8-H8	21.4	20.2
G21	C1'-H1'	-41.8	NA
G21	C8-H8	21.3	22.5
G21	N1-H1	-11	NA
A22	C1'-H1'	-1.5	-2.9
A22	C2-H2	22	23.2
A22	C8-H8	22.5	NA
U23	C1'-H1'	3.6	1.8
U23	C5-H5	10.2	12.3
U23	C6-H6	9.7	9
C24	C6-H6	26	NA
U25	C1'-H1'	-25.7	NA
U25	C6-H6	24.5	NA
U25	N3-H3	-14.2	-14.7
G26	C8-H8	32.4	29.5
G26	N1-H1	-14.5	NA
A27	C1'-H1'	-0.1	NA
A27	C2-H2	27.9	NA
A27	C8-H8	29.6	25.1
G28	C1'-H1'	-35.8	NA
C29	C6-H6	21.3	NA
C30	C1'-H1'	-18.6	NA
G33	C8-H8	30.6	28.2
G33	N1-H1	-10.3	-15.2
G34	C8-H8	28	31.6
A35	C1'-H1'	-6.4	-3.9
A35	C2-H2	26.5	29.3
G36	C1'-H1'	-29	NA
G36	C8-H8	30.6	29.7
C37	C5-H5	24.4	NA
U38	C6-H6	27.3	NA
U38	N3-H3	-12.1	-12.4
C39	C1'-H1'	-21.7	NA
U40	C6-H6	23.8	NA
C41	C6-H6	16	NA
U42	C6-H6	16.6	NA
U42	N3-H3	-9.2	NA
G43	C1'-H1'	-16.2	NA
G43	C8-H8	18.9	19.4

Supplementary Table 2. List of representative tandem AG mismatches in the PDB structure survey. The complete list is available on Github. Base pair geometries are represented using the Leontis–Westhof[10] (LW) classification. (e.g. "cWW" denotes the A-G bp as *cis* and hydrogen-bonded using the Watson–Crick:Watson–Crick edge).

pdbid	uniprot	metal	LW	residue_1	residue_2	base pair environment
1vy5	Ribosome	MG | ZN | K	cWS	1:BA.A699	1:BA.G1633	1. Located at turn-loop like structural motif; 2. Not adjacent to any bps
			cSS	1:DA.G700	1:DA.A1632	
1vy6		MG | ZN | K	tHS	1:BA.A84	1:BA.G102	1. Located at turn-loop like structural motif; 2.A84 forms bps with more than 1 complementary residues; 3. Adjacent to G-U mismatch; 4. In the context of 5'GAG/3'AGA
			tSW	1:DA.G85	1:DA.A103	
3cma		MG | SR | NA | CD | K	tHS	1:0.A939	1:0.G1031	1. Located at turn-loop like structure; 2. In the context of 5'-GAG/3'AGA
			tSS	1:0.G940	1:0.A1032	
4u1u		MG | ZN	cWH	1:DA.A2531	1:DA.G2661	1. Located at turn-loop like structural motif; 2. A2662 forms bps with more than 1 complementary residues
			tWW	1:DA.G2532	1:DA.A2662	
4u4r		MG | OHX | ZN	tHS	1:6.A898	1:6.G914	1. Adjacent to the AC mismatch; 2.G899 forms bps with more than 1 complementary residues
			tS.	1:2.G899	1:2.A915	
5ndk		MG | ZN	cWS	1:14.A748	1:14.G1683	1. Two apical loop like structures; 2. No additional contact except the formation of tandem AG
			cSS	1:14.G749	1:14.A1682	
			tHS	1:1G.A887	1:1G.G922	
			tSS	1:1G.G888	1:1G.A923	
3t1y		MG | ZN	tHS	1:A.A241	1:A.G276	Adjacent to flipped out residues
			tHS	1:A.A670	1:A.G686	
5hd1		MG | ZN	tHS	1:1a.A242	1:1a.G277	Adjacent to flipped out residues
			tSS	1:2a.G243	1:2a.A278	
			tHS	1:1a.A671	1:1a.G687	1. Adjacent to flipped out residues; 2. Both A671 and G672 form bps with more than 1 complementary residues
			tSS	1:1a.G672	1:1a.A688	
4y1o	Transferase	MG | K	cWW	1:B.A159	1:B.G218	Adjacent to flipped out residues
			cWW	1:B.G160	1:B.A217	
4aob	SAM-I riboswitch	BA | K | NA	tHS	1:A.A20	1:A.G35	1. Adjacent to CC mismatches; 2. Both A20 and G21 form bps with more than 1 complementary residues
			tSS	1:A.G21	1:A.A36	
3rw6	Nuclear RNA export factor 1		tHS	1:F.A17	1:F.G47	1.Adjacent to CC mismatches and protein interaction site; 2.G18 forms bps with more than 1 complementary residues
			tSS	1:H.G18	1:H.A48	

Supplementary Table 3. Exchange parameters obtained from 2-state fitting of ^{1}H CEST, ^{15}N CEST, and ^{15}N $R_{1\rho}$ data.

Sample	Resonance	p_{ES} (%)	k_{ex} (s^{-1})	Δω (ppm)	R_1 (s^{-1})	R_2 (s^{-1})	Red. rχ^2
^{1}H CEST							
wtTAR 25°C, T_{ex} = 100 ms	Shared 2-state Fitting						
	U38-H3	0.25±0.01	737±39	-2.85±0.02	9.27±0.01	27.23±0.13	13.51
	G28-H1			-0.76±0.01	5.44±0.0	25.31±0.07	
	G36-H1			-0.39±0.01	7.14±0.0	28.69±0.06	
	G26-H1			0.41±0.07	11.06±0.02	31.43±0.27	
	Individual Fitting						
	U38-H3	0.4±0.04	473±46	-2.84±0.01	9.25±0.01	27.18±0.09	5.16
	G28-H1	0.28±0.02	510±62	-0.74±0.01	5.43±0.0	25.46±0.09	19.91
	G36-H1	0.24±0.06	320±121	-0.45±0.02	7.14±0.0	28.85±0.08	21.28
	G26-H1	0.57±0.11	329±110	0.33±0.02	11.06±0.01	31.44±0.14	3.5
wtTAR 30°C, T_{ex} = 80 ms	U38-H3	0.36±0.04	1006.49±79.43	-2.81±0.01	13.07±0.01	28.3±0.16	13.86
wtTAR 35°C, T_{ex} = 80 ms	U38-H3	0.26±0.02	2369.35±156.07	-2.81±0.03	20.03±0.02	33.99±0.28	1.26
wtTAR 40°C, T_{ex} = 80ms	U38-H3	0.24±0.04	4417.05±1044.8	-2.73±0.17	31.09±0.07	37.97±1.07	6.20
TAR^{ES2} 25°C, T_{ex} = 80 ms	U38-H3	14.71±8.01	160.2±12.26	2.93±0.01	11.76±0.01	33.73±0.84	31.52
TAR^{ES2} 30°C, T_{ex} = 50 ms	U38-H3	18.96±21.86	272.9±19.44	2.93±0.0	19.41±0.03	39.88±1.35	5.62
G36U 15°C, T_{ex} = 80 ms	U38-H3	1.59±0.43	221.82±40.82	3.03±0.01	7.17±0.01	39.32±0.31	4.89
G36U 20°C, T_{ex} = 80 ms	U38-H3	2.52±0.69	462.1±19.66	3.04±0.0	10.5±0.01	40.79±0.68	5.10
G36U 25°C, T_{ex} = 100 ms	U38-H3	3.67±2.34	853.4±71.1	3.04±0.01	16.04±0.03	46.01±2.65	3.43
^{15}N CEST							
wtTAR 25°C, T_{ex} = 300 ms	U38-N3	0.31±0.03	316±25	-5.10±0.05	2.63±0.00	8.28±0.11	5.34
wtTAR 30°C, T_{ex} = 300 ms	U38-N3	0.25±0.02	836±67	-5.04±0.12	2.89±0.00	7.75±0.15	3.89
^{15}N $R_{1\rho}$							
wtTAR 35°C	U38-N3	0.23±0.01	1673±85	-5.00±0.13	1.87±0.01	5.69±0.04	0.34

Supplementary Table 4. Exchange parameters obtained from 3-state fitting of ^{1}H CEST data for c^7A wtTAR and c^7A 2U.

**Sample**	**c^7A wtTAR 25°C, pH 6.4, T_{ex} = 80ms**				**c^7A 2U 25°C, pH 6.4, T_{ex} = 80ms**			
**Fitting**	Shared 3-state Fitting		Individual Fitting		Shared 3-state Fitting		Individual Fitting	
**Resonance**	G28	G26	G28	G26	G28	G26	G28	G26
**p_B (%)**	1.69±0.1		1.73±0.08	1.68±1.06	1.52±0.17		1.18±0.46	1.94±0.28
**p_C (%)**	0.29±0.02	N/A	0.29±0.02	N/A	0.19±0.06	0.53±0.08	0.20±0.09	0.54±0.10
**$\Delta\omega_B$ (ppm)**	-0.68±0.01	0.51±0.01	-0.68±0.01	0.52±0.01	-0.50±0.04	0.40±0.02	-0.31±0.17	0.38±0.02
**$\Delta\omega_C$ (ppm)**	-2.57±0.03	N/A	-2.58±0.02	N/A	-2.57±0.09	-2.28±0.33	-2.24±0.26	-2.27±0.36
**$k_{ex}AB$ (s^{-1})**	614±52		657±48	556±116	928±177		757±609	1152±271
**$k_{ex}AC$ (s^{-1})**	149±192	N/A	1.0±142	N/A	1.7±557	13884±3155	45±2781	13703±3457
**$k_{ex}BC$ (s^{-1})**	1908±305	N/A	2074±237	N/A	2294±1093	1.1±522	10991±4487	1±594
**R_1 (s^{-1})**	11.27±0.02	20.06±0.02	11.27±0.01	20.05±0.02	10.39±0.01	14.45±0.02	10.39±0.01	14.46±0.02
**R_2 (s^{-1})**	40.84±0.48	56.53±0.65	40.77±0.37	56.96±0.82	34.97±0.51	33.43±5.09	33.94±1.07	30.14±5.52
**Red. $r\chi^2$**	4.15		2.42	5.89	20.06		13.75	26.5

Supplementary Table 5. List of RF powers (ω_1 $2\pi^{-1}(s^{-1})$) and offsets (Ω $2\pi^{-1}(s^{-1})$) used in the CEST experiments.

**T (°C)**	**ω_1 $2\pi^{-1}$ (s^{-1})**	**Ω $2\pi^{-1}(s^{-1})$**
^{1}H CEST wtTAR (pH 6.4, 90% H_2O:10% D_2O)		
25 °C,<br>T_{ex} = 100 ms	10<br>50<br>100<br>200<br>500<br>1000<br>4000	[-4581, -4324, -4067, -3810, -3553, -3296, -3039, -2782, -2722, -2662, -2602, -2542, -2482, -2422, -2362, -2302, -2242, -2182, -2122, -2063, -2003, -1943, -1883, -1823, -1763, -1703, -1643, -1583, -1523, -1463, -1403, -1343, -1283, -1223, -1163, -1103, -1043, -983, -923, -863, -803, -743, -683, -623, -563, -503, -443, -383, -324, -264, -204, -84, 95, 155, 215, 275, 335, 395, 455, 515, 575, 635, 695, 755, 815, 875, 935, 995, 1055, 1115, 1175, 1235, 1295, 1355, 1415, 1474, 1534, 1594, 1654, 1714, 1774, 1834, 1894, 1954, 2014, 2271, 2528, 2785, 3042, 3299, 3556, 3813]
30 °C,<br>T_{ex} = 80 ms	10<br>50<br>100<br>200<br>500<br>1000<br>4000	[-3685, -3385, -3085, -2786, -2486, -2411, -2336, -2261, -2186, -2111, -2036, -1961, -1886, -1811, -1736, -1661, -1586, -1511, -1436, -1361, -1286, -1211, -1137, -1062, -987, -912, -837, -762, -687, -616, -546, -475, -405, -334, -264, -193, -122, 88, 159, 229, 300, 370, 441, 512, 586, 661, 736, 811, 886, 961, 1036, 1111, 1186, 1261, 1336, 1411, 1486, 1561, 1636, 1711, 1786, 1861, 1936, 2011, 2086, 2161, 2236, 2311, 2610, 2910, 3210, 3510]
35 °C,<br>T_{ex} = 80 ms	10<br>50<br>100<br>200<br>500<br>1000<br>4000	[-4626, -4369, -4112, -3855, -3598, -3341, -3084, -2827, -2767, -2708, -2648, -2588, -2528, -2468, -2408, -2348, -2288, -2228, -2168, -2108, -2048, -1988, -1928, -1868, -1808, -1748, -1688, -1628, -1568, -1508, -1448, -1388, -1328, -1268, -1208, -1148, -1088, -1028, -969, -909, -849, -789, -729, -669, -609, -549, -489, -429, -369, -309, -249, -189, -129, -69, -9, 50, 110, 170, 230, 290, 350, 410, 470, 530, 590, 650, 710, 770, 829, 889, 949, 1009, 1069, 1129, 1189, 1249, 1309, 1369, 1429, 1489, 1549, 1609, 1669, 1729, 1789, 1849, 1909, 1969, 2226, 2483, 2740, 2997, 3254, 3511, 3768]
40 °C,<br>T_{ex} = 80 ms	10<br>50<br>200<br>500<br>1000<br>4000	[-4176, -3919, -3662, -3405, -3148, -2891, -2634, -2377, -2317, -2257, -2197, -2137, -2077, -2017, -1957, -1897, -1837, -1777, -1717, -1657, -1597, -1537, -1477, -1417, -1357, -1297, -1237, -1177, -1117, -1057, -997, -937, -877, -817, -757, -698, -638, -578, -518, -458, -398, -338, -278, -218, -158, -98, -38, 21, 81, 141, 201, 261, 321, 381, 441, 501, 561, 621, 681, 741, 801, 861, 921, 981, 1041, 1100, 1160, 1220, 1280, 1340, 1400, 1460, 1520, 1580, 1640, 1700, 1760, 1820, 1880, 1940, 2000, 2060, 2120, 2180, 2240, 2300, 2360, 2420, 2677, 2934, 3191, 3448, 3705, 3962, 4219]
^{15}N CEST, wtTAR (pH 6.4, 90% H_2O:10% D_2O)		
25 °C,<br>T_{ex} = 300 ms	10	[-800.0, -792.965, -785.93, -778.894, -771.859, -764.824, -757.789, -750.754, -743.719, -736.683, -729.648, -722.613, -715.578, -708.543, -

	20	701.508, -694.472, -687.437, -680.402, -673.367, -666.332, -659.296, -652.261, -645.226, -638.191, -631.156, -624.121, -617.085, -610.05, -603.015, -595.98, -588.945, -581.91, -574.874, -567.839, -560.804, -553.769, -546.734, -539.698, -532.663, -525.628, -518.593, -511.558, -504.523, -497.487, -490.452, -483.417, -476.382, -469.347, -462.312, -455.276, -448.241, -441.206, -434.171, -427.136, -420.101, -413.065, -406.03, -398.995, -391.96, -384.925, -377.889, -370.854, -363.819, -356.784, -349.749, -342.714, -335.678, -328.643, -321.608, -314.573, -307.538, -300.503, -293.467, -286.432, -279.397, -272.362, -265.327, -258.291, -251.256, -244.221, -237.186, -230.151, -223.116, -216.08, -209.045, -202.01, -194.975, -187.94, -180.905, -173.869, -166.834, -159.799, -152.764, -145.729, -138.693, -131.658, -124.623, -117.588, -110.553, -103.518, -96.482, -89.447, -82.412, -75.377, -68.342, -61.307, -54.271, -47.236, -40.201, -33.166, -26.131, -19.095, -12.06, -5.025, 2.01, 9.045, 16.08, 23.116, 30.151, 37.186, 44.221, 51.256, 58.291, 65.327, 72.362, 79.397, 86.432, 93.467, 100.503, 107.538, 114.573, 121.608, 128.643, 135.678, 142.714, 149.749, 156.784, 163.819, 170.854, 177.889, 184.925, 191.96, 198.995, 206.03, 213.065, 220.101, 227.136, 234.171, 241.206, 248.241, 255.276, 262.312, 269.347, 276.382, 283.417, 290.452, 297.487, 304.523, 311.558, 318.593, 325.628, 332.663, 339.698, 346.734, 353.769, 360.804, 367.839, 374.874, 381.91, 388.945, 395.98, 403.015, 410.05, 417.085, 424.121, 431.156, 438.191, 445.226, 452.261, 459.296, 466.332, 473.367, 480.402, 487.437, 494.472, 501.508, 508.543, 515.578, 522.613, 529.648, 536.683, 543.719, 550.754, 557.789, 564.824, 571.859, 578.894, 585.93, 592.965, 600.0]
	40	
	50	
	100	
30 °C, T_{ex} = 300 ms	10	
	20	
	40	
	50	
	100	
[1]H CEST, G36U (pH 6.4, 90% H_2O:10% D_2O)		
15 °C, T_{ex} = 80 ms	10	[-5391, -4941, -4491, -4041, -3591, -3483, -3375, -3267, -3159, -3051, -2943, -2835, -2727, -2619, -2511, -2403, -2295, -2187, -2079, -1971, -1863, -1755, -1647, -1539, -1431, -1323, -1215, -1107, -999, -891, -785, -679, -573, -467, -362, -256, -150, 61, 167, 273, 379, 485, 590, 696, 802, 908, 1016, 1124, 1232, 1340, 1448, 1556, 1664, 1772, 1880, 1988, 2096, 2204, 2312, 2420, 2528, 2636, 2744, 2852, 2960, 3068, 3176, 3284, 3392, 3500, 3608, 4058, 4508, 4958, 5408]
	50	
	100	
	500	
20 °C, T_{ex} = 80 ms	10	[-5376, -4926, -4476, -4026, -3576, -3468, -3360, -3252, -3144, -3036, -2928, -2820, -2712, -2604, -2496, -2388, -2280, -2172, -2064, -1956, -1848, -1740, -1632, -1524, -1416, -1308, -1200, -1092, -984, -876, -770, -665, -559, -453, -347, -241, -135, -29, 76, 182, 287, 393, 499, 605, 711, 817, 923, 1031, 1139, 1247, 1355, 1463, 1571, 1679, 1787, 1895, 2003, 2111, 2219, 2327, 2435, 2543, 2651, 2759, 2867, 2975, 3083, 3191, 3299, 3407, 3515, 3623, 4073, 4523, 4973, 5423]
	50	
	100	
	500	
25 °C, T_{ex} = 100 ms	10	[-5388, -4938, -4488, -4038, -3588, -3480, -3372, -3264, -3156, -3048, -2940, -2832, -2724, -2616, -2508, -2400, -2292, -2184, -2076, -1968, -1860, -1752, -1644, -1536, -1428, -1320, -1212, -1104, -996, -888, -782, -676, -570, -464, -358, -253, -41, 64, 276, 382, 488, 593, 699, 805, 911, 1019, 1127, 1235, 1343, 1451, 1559, 1667, 1775, 1883, 1991, 2099, 2207, 2315, 2423, 2531, 2639, 2747, 2855, 2963, 3071, 3179, 3287, 3395, 3503, 3611, 4061, 4511, 4961, 5411]
	50	
	100	
	200	
	500	
[1]H CEST, c^7A wtTAR (pH 6.4, 90% H_2O:10% D_2O)		

25 °C, T_{ex} = 80 ms	10	[-5391, -4941, -4491, -4041, -3591, -3483, -3375, -3267, -3159, -3051, -2943, -2835, -2727, -2619, -2511, -2403, -2295, -2187, -2079, -1971, -1863, -1755, -1647, -1539, -1431, -1323, -1215, -1107, -999, -891, -785, -679, -573, -467, -362, -256, -150, 61, 167, 273, 379, 485, 590, 696, 802, 908, 1016, 1124, 1232, 1340, 1448, 1556, 1664, 1772, 1880, 1988, 2096, 2204, 2312, 2420, 2528, 2636, 2744, 2852, 2960, 3068, 3176, 3284, 3392, 3500, 3608, 4058, 4508, 4958, 5408]
	50	
	100	
	200	
	500	
	1000	
	4000	
^1H CEST, c^7A 2U (pH 6.4, 90% H_2O:10% D_2O)		
25 °C, T_{ex} = 80 ms	10	[-6639, -6189, -5739, -5288, -4838, -4388, -3938, -3830, -3722, -3614, -3506, -3398, -3290, -3182, -3074, -2966, -2858, -2750, -2642, -2534, -2426, -2318, -2210, -2102, -1994, -1886, -1778, -1670, -1562, -1454, -1346, -1238, -1132, -1027, -921, -815, -709, -603, -497, -391, -285, -179, -74, 31, 137, 243, 349, 455, 561, 669, 777, 885, 993, 1101, 1209, 1317, 1425, 1533, 1641, 1749, 1857, 1965, 2073, 2181, 2289, 2397, 2505, 2613, 2721, 2829, 2937, 3045, 3153, 3261, 3711, 4161, 4611, 5061, 5511, 5961]
	50	
	100	
	200	
	500	
	1000	

Supplementary Table 6. List of spin-lock power (ω_1 $2\pi^{-1}(s^{-1})$) and offsets (Ω $2\pi^{-1}(s^{-1})$) used in the $R_{1\rho}$ experiments.

**Nucleus**	**[ω_1 $2\pi^{-1}(s^{-1})$] [Ω $2\pi^{-1}(s^{-1})$]**
wtTAR U38-N3 (pH 6.4, 35 °C, 90% H_2O:10% D_2O)	[150] [-532, -494, -456, -418, -380, -342, -304, -266, -228, -190, -152, -114, -76, -38, -10, 10, 38, 76, 114, 152, 190, 228, 266, 304, 342, 380, 418, 456, 494, 532]
	[200] [-700, -650, -600, -550, -500, -450, -400, -350, -300, -250, -200, -150, -100, -50, -10, 10, 50, 100, 150, 200, 250, 300, 350, 400, 450, 500, 550, 600, 650, 700]
	[300] [-1050, -975, -900, -825, -750, -675, -600, -525, -450, -375, -300, -225, -150, -75, -10, 10, 75, 150, 225, 300, 375, 450, 525, 600, 675, 750, 825, 900, 975, 1050]
	[400] [-1400, -1300, -1200, -1100, -1000, -900, -800, -700, -600, -500, -400, -300, -200, -100, -10, 10, 100, 200, 300, 400, 500, 600, 700, 800, 900, 1000, 1100, 1200, 1300, 1400]
	[600] [-2100, -1950, -1800, -1650, -1500, -1350, -1200, -1050, -900, -750, -600, -450, -300, -150, -10, 10, 150, 300, 450, 600, 750, 900, 1050, 1200, 1350, 1500, 1650, 1800, 1950, 2100]
	[1000] [-3500, -3250, -3000, -2750, -2500, -2250, -2000, -1750, -1500, -1250, -1000, -750, -500, -250, -10, 10, 250, 500, 750, 1000, 1250, 1500, 1750, 2000, 2250, 2500, 2750, 3000, 3250, 3500]
List of delay (ms): 0, 40, 80, 120, 160	

Supplementary Table 7. Secondary structure and base pair geometry constraints used to generate the TAR^{ES2} FARFAR library.

2º structure	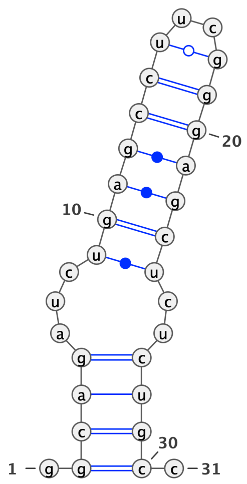
Input files	input.fasta:<br>> *uucg-tar-es2*<br>*ggcagaucugagccuucgggagcucucugcc*<br>input.secstruct:<br>*.((((...[([[(([..]))]])]..)))).*
obligate bp constrains	*BasePair 9 24 W W C* (U25-U38 Watson-Crick bp)
Command	*rna_denovo.linuxgccrelease -secstruct_general_file *.secstruct_general -nstruct 250 -fasta *.fasta -s imino_helix_* -obligate_pair_explicit 15 18 S W T -cst_file *.cst -use_legacy_job_distributor -minimize_rna true -score:weights stepwise/rna/rna_res_level_energy4.wts -restore_talaris_behavior -out:file:silent *.out*

Supplementary Movie 1. The FARFAR-NMR atomic-resolution dynamic ensemble of HIV-1 TARES2. Structural motifs are color-coded according to Fig. 3(a)

References

1. Dethoff, E.A. et al. Characterizing complex dynamics in the transactivation response element apical loop and motional correlations with the bulge by NMR, molecular dynamics, and mutagenesis. *Biophys J* **95**, 3906-3915 (2008).
2. Clay, M.C., Ganser, L.R., Merriman, D.K. & Al-Hashimi, H.M. Resolving sugar puckers in RNA excited states exposes slow modes of repuckering dynamics. *Nucleic Acids Res* **45**, e134-e134 (2017).
3. Lee, J., Dethoff, E.A. & Al-Hashimi, H.M. Invisible RNA state dynamically couples distant motifs. *Proc Natl Acad Sci U S A* **111**, 9485-9490 (2014).
4. Merriman, D.K. et al. Shortening the HIV-1 TAR RNA Bulge by a Single Nucleotide Preserves Motional Modes over a Broad Range of Time Scales. *Biochemistry* **55**, 4445-4456 (2016).
5. Rangadurai, A., Szymaski, E.S., Kimsey, I.J., Shi, H. & Al-Hashimi, H.M. Characterizing micro-to-millisecond chemical exchange in nucleic acids using off-resonance R1ρ relaxation dispersion. *Prog Nucl Magn Reson Spectrosc* **112-113**, 55-102 (2019).
6. Santa Lucia, J., Jr. & Turner, D.H. Structure of (rGGCGAGCC)2 in solution from NMR and restrained molecular dynamics. *Biochemistry* **32**, 12612-12623 (1993).
7. Heus, H.A., Wijmenga, S.S., Hoppe, H. & Hilbers, C.W. The detailed structure of tandem G·A mismatched base-pair motifs in RNA duplexes is context dependent11Edited by I. Tinoco. *J Mol Biol* **271**, 147-158 (1997).
8. Nikolova, E.N., Gottardo, F.L. & Al-Hashimi, H.M. Probing Transient Hoogsteen Hydrogen Bonds in Canonical Duplex DNA Using NMR Relaxation Dispersion and Single-Atom Substitution. *J Am Chem Soc* **134**, 3667-3670 (2012).
9. Kimsey, I.J., Petzold, K., Sathyamoorthy, B., Stein, Z.W. & Al-Hashimi, H.M. Visualizing transient Watson–Crick-like mispairs in DNA and RNA duplexes. *Nature* **519**, 315-320 (2015).
10. Leontis, N.B., Stombaugh, J. & Westhof, E. The non-Watson–Crick base pairs and their associated isostericity matrices. *Nucleic Acids Res* **30**, 3497-3531 (2002).
