# Supplementary material for: An RNA excited conformational state at atomic resolution
Source: Nat Commun. 2023 Dec 19;14:8432. doi: 10.1038/s41467-023-43673-6 (PMC10730710; doi:10.1038/s41467-023-43673-6)
Supplement: Supplementary file 3 — Description of Additional Supplementary Information [file 41467_2023_43673_MOESM3_ESM.pdf]

Description of Additional Supplementary Files

File name: Supplementary Movie 1

Description: The FARFAR-NMR atomic-resolution dynamic ensemble of HIV-1 TARES2. Structural motifs are color-coded according to Fig. 3(a).
